# Comparison of external load measures per minute based on match seasonal periods and playing positions of a soccer Turkish super league team

**DOI:** 10.1186/s12891-023-06787-y

**Published:** 2023-08-17

**Authors:** Zeki Akyildiz, Yılmaz Yüksel, Yaşar Birgonül, Halil İbrahim Ceylan, Rafael Oliveira, Erhan Çene, Coskun Parim, Filipe Manuel Clemente, Hadi Nobari

**Affiliations:** 1 Sports Science Department, Gazi University, Ankara, 06570 Turkey; 2 Sports Science Department, Anadolu University, Eskisehir, 26170 Turkey; 3 Physical Education and Sports Teaching Department, Kazim Karabekir Faculty of Education, Ataturk University, Erzurum, 25030 Turkey; 4 Research Centre in Sports Sciences, Health Sciences and Human Development (CIDESD), Vila Real, 5001-801 Portugal; 5 Sports Science School of Rio Maior, Polytechnic Institute of Santarém, Rio Maior, 2040-413 Portugal; 6 Life Quality Research Centre, Rio Maior, 2040-413 Portugal; 7 Department of Statistics, Yildiz Technical University, Istanbul, 34220 Turkey; 8 Escola Superior Desporto e Lazer, Instituto Politécnico de Viana do Castelo, Rua Escola Industrial e Comercial de Nun’Álvares, Viana do Castelo, 4900-347 Portugal; 9 Delegação da Covilhã, Instituto de Telecomunicações, Lisboa, 1049-001 Portugal; 10 Department of Exercise Physiology, Faculty of Educational Sciences and Psychology, University of Mohaghegh Ardabili, Ardabil, 5619911367 Iran; 11 Faculty of Sport Sciences, University of Extremadura, Cáceres, 10003 Spain

**Keywords:** Match Analysis, External load, Playing positions, Seasonal periods, Soccer

## Abstract

**Background:**

Turkish Super League teams need more information about the external load. Considering the specific country and the coaches’ philosophies, the purpose of this study was to compare the external match load of a Turkish Super League team considering the different playing positions and in-season periods.

**Methodology:**

A longitudinal study design was employed by observing 29 official matches of the same team. A total of fifteen players, consisting of five defenders, five midfielders, and five forwards, were analyzed using the Sentio Sports Optical Tracking System. The following outcomes were extracted in each match: total distance (TD), walking [from 0 to 7.2 km/h], jogging [from 7.2 to 14.4 km/h], running [from 14.4 to 20 km/h], high speed running (HSR) [> 20 km/h], metabolic power (MP), maximum deceleration (Dec_max_) [Dec < -3 m/s^2^], total deceleration distance (Dec_total_), maximum acceleration (Acc_max_) [Acc > 3 m/s^2^], and total acceleration distance (Acc_total_). Statistical analysis consisted of ANOVA and Bonferroni correction post hoc tests.

**Results:**

The main results showed that all variables were similar between periods of the season (p > 0.05) except for walking (p = 0.021 witha large effect size), which revealed lower values in the mid-season period. When analyzing periods of the season considering playing positions, there were several significant results for TD, walking, jogging, running, HSR, Dec_total_ and Acc_total_ (p < 0.05, with small to large effects). A tendency for higher values was noted in the mid and end-season. Considering the playing positions comparisons, midfielders showed higher values than defenders for TD, MP, Dec_total_ and Acc_total_ (p < 0.05 with large effect for all). Midfielders also showed higher values than forwards for TD, jogging, Dec_total_ and Acc_total_ (p < 0.05 with a large effect for all).

**Conclusions:**

The present study emphasizes the significance of analyzing data based on minute, playing position, and season period. Findings reveal that defenders consistently displayed the lowest values in all external load measures during matches compared to other positions throughout the season. Furthermore, midfielders demonstrated a higher activity profile during the initial and middle stages of the season compared to other positions, with a slight tendency to decrease load towards the end of the season.

## Introduction

Competitive soccer matches presented several intermittent high-intensity and low-intensity. While total distance describes volume of the session, other measures such as sprint distances, accelerations, and/or decelerations are involved in the actions related to the decisive moments of a soccer match. They can determine the load of the training process [1]. However, these intense actions may provoke neuromuscular fatigue and a possible high risk of injury [2].

The knowledge of the activities matches produced is fundamental for better load and intensity monitoring [3]. In this sense, there are some equipment such as global positioning systems, telemetry and micro technologies (wearable inertial measurement units and electronic performance tracking systems) that were developed to access external demands in order to help coaches and their staff monitoring practices [4].

Recently, an updated concept was provided to describe external load monitoring that consists in the physical demands imposed by the design and mode of exercise [5]. For better clarity of concepts, these authors referred to external activities as one dimension of load monitoring.

External load monitoring in matches has revealed that midfielders covered a higher total distance than defenders and forwards during matches [6]. Recently, it was shown that wide defenders achieved 64% while central defenders, central midfielders, and central forwards achieved 107%, 100%, and 107%, respectively, in training load compared to matches [7]. Moreover, it was also shown that different speed thresholds, player load, accelerations and decelerations were significantly different according to player positions during matches [8]. For instance, central defenders showed the lowest values while wide defenders and midfielders show the highest.

As mentioned before, soccer matches encompass a wide range of physically demanding performance characteristics. For instance, it was reported the total distance covered during the match was in the range of ∼8–11 km, while the relative distance was in the range of ∼100–120 m/min per match in elite soccer players [8–10]. Moreover, soccer matches present several high-intensity actions that include high-speed running, accelerations/ decelerations and consequently a great metabolic load even with lower intensities [11]. In this regard, the high-speed running distance in a match is around 8% of the total distance covered [12]. In addition, the number of accelerations and deceleration belong to a large portion of the match, affecting biomechanical and physiological players [2]. For instance, it was shown that a professional Norwegian team performed 7–10% of accelerations and 5–7% of decelerations of the total player load for all playing positions in the match (with the exception of goalkeepers) [8].

Furthermore, the quantification of external load during matches allows a better understanding of the season variations. As an example, the analysis of three periods (early-, mid- and end-season) showed several load fluctuations during the season [13]. However, the previous study used specific indexes (e.g. monotony and strain) that included calculations with data from both training and match sessions [13] and for that reason, it is difficult to understand the variation regarding the match data values. An easy way to fix this issue could be to use of the “m/s” unit when evaluating the different running distances at different speeds in to the detriment of “km/h” as previously suggested [3].

Considering that only one study was found to analyze external load in matches from professional Turkish players, but it did not analyze playing positions and the differences between periods of the season [14], it is necessary to conduct more studies that better contextualize the specific context of professional Turkish players. Besides, the previous research presented in this introduction could not be representative of the usual external demands from other teams of other countries due to the different coaches philosophies. Therefore, the aim of this study was to compare the external match load of a Turkish Super League team considering the different playing positions and in-season periods. It was hypothesized that several fluctuations could occur across the season [13]. It was also hypothesized that external load would vary among playing positions [6–8].

## Materials and methods

### Study design and experimental approach

The data analyzed in this study belong to an elite soccer team in the Turkish Super League which is the first division in Turkey. Elite athletes in the team trained 6 days a week with one match per week. The participants involved in this research are professional soccer players who compete in the Turkish Super League of their respective country. These players possess a minimum of 5 years of experience in the field of soccer. The dataset comprises performance metrics of soccer players at the individual match level during the 2019–2020 season of a Turkish Super League team. The dataset encompasses a total of 26 league matches and 3 continental cup matches, all of which were held between August 2019 and July 2020. These matches are categorized into three distinct cycles, namely the start of the competitive season (consisting of 13 matches spanning from August 2019 to November 2019), the mid-competitive season (comprising 11 matches held between December 2019 and March 2020), and the end of the competitive season (encompassing 5 matches played from June 2020 to July 2020). The matches played every weekend were recorded with the Sentio Sports optical tracking system. This study was approved by the (Atatürk University Faculty of Sports Sciences Ethics committee approval number: e-70400699-050.02.04-2300158950). The entire work follows the Declaration of Helsinki for the Humanities.

### Data collection and measurement

All the data of the team were collected through optical cameras, namely, the Sentio Sports optical tracking system that consists of two cameras with 4 K resolution, a notebook and a Sentio Scope software. Previous studies have reported that the Sentio system offers valid and reliable data [22–25]. After the cameras were connected to the computer, Sentio software made the sharpness adjustment and calibration on the field image of the cameras and controlled the obtained data. After the device and software installation, a technician provided instant control to get the data. To minimize the margin of error in corners and crown points, the technicians instantly controlled the data flow. The cameras are securely positioned within the live broadcast room by the broadcaster, specifically at the midfield line level, allowing for a comprehensive view of the field in two sections. Once the cameras are connected to the computer, the Sentio software facilitates the adjustment of image sharpness and camera calibration on the field. During calibration, the software prompts the user to define a specific number of points as requested by the system.

Once the team staff is encoded into the Scope software by an operator, the system automatically initiates player tracking and records the positional data of each player. Due to the proximity of players to one another during corner kicks and set-piece situations, the system does not assign location data to individual players. Consequently, the operator takes on the responsibility of identifying the players during such instances to ensure correct player attribution and prevent data loss. The Scope software periodically prompts the operator with location-based questions to maintain the accuracy of the optical tracking throughout the match.

The dataset contains soccer players’ individual match performance metrics of the 2019–2020 season from a Turkish Super League team. Data contains 26 league and three continental cup matches that take place between August 2019 and July 2020. Available matches are divided into three cycles named as the start of the competitive season (13 matches – from August 2019 to November 2019), mid of the competitive season (11 matches – from December 2019 to March 2020) and end of the competitive season (5 matches – from June 2020 to July 2020) [15]. During April and May, there was disruption of training sessions and matches a due to the COVID-19 pandemic.

Only outfield players who participated in all three cycles and played at least 45 min in a match are considered in the dataset. Data has been standardized by dividing the performance metrics to playing time to eliminate the effect of differences in the playing time. After these arrangements dataset contains 15 unique players (five defenders, five midfielders, and five forwards) with a total of 250 individual match observations. The mean and standard deviation of age, height and body weight for the players are 30.58 ± 4.03 years, 1.82 ± 0.07 m, and 76.75 ± 7.53 kg, respectively.

Total distance (TD), walking [from 0 to 7.2 km/h], jogging [from 7.2 to 14.4 km/h], running [from 14.4 to 20 km/h], high speed running (HSR) [> 20 km/h], metabolic power (MP) (W/kg), maximum deceleration (Dec_max_) [Dec< -3 m/s^2^] [15], total deceleration distance (Dec_total_), maximum acceleration (Acc_max_) [Acc > 3 m/s^2^], and total acceleration distance (Acc_total_), are the considered variables in the analysis.

### Statistical analysis

The normality of the variables is checked with the Shapiro-Wilk test, and it is seen that all variables follow a normal distribution. For each cycle, the mean and standard deviation for performance metrics is reported among player positions. Differences in the performance metrics for each playing position among early season, mid-season, and late-season (Table 1) and the differences in the performance metrics for each season cycle among player positions (Table 1) is checked with a Mixed ANOVA test. In case of detecting any significant differences, Bonferroni post hoc test is employed to find the source of difference. Effect size (${\eta }^{2}$) values and Cohen’s d values (d) are also reported. ${\eta }^{2}$ values in the range 0-0.009 are considered insignificant effect sizes, 0.01-0.0588 as small effect sizes, 0.0589–0.1379 as medium effect sizes, and values greater than 0.1379 as large effect sizes [16]. Percentage changes among season cycles and positions are also reported.

**Table 1:** Mean, Std. Deviations and Mixed ANOVA results for match activities according to season periods for all players combined

Variables (per min)	Start	Mid		End		F	p	Effect Size	Source of Diff.
	Mean ± SD	Mean ± SD	%Change	Mean ± SD	%Change				
TD (m)	105.63 ± 7.58	106.05 ± 8.69	0.40%	103.19 ± 6.45	-2.70%	0.494	0.615	0.029 (Small)	----
Walking	42.08 ± 1.81	40.2 ± 1.84	-4.47%	42.28 ± 2.06	5.18%	4.361	0.021	0.209 (Large)	Start > Mid; End > Mid
Jogging	41.13 ± 3.82	42.88 ± 4.08	4.27%	39.56 ± 3.48	-7.74%	2.291	0.117	0.122 (Medium)	----
Running	15.19 ± 3.78	16.58 ± 3.86	9.17%	14.41 ± 3.26	-13.08%	1.09	0.348	0.062 (Medium)	----
HSR	7.21 ± 1.98	7.38 ± 2	2.38%	6.91 ± 2.01	-6.41%	0.173	0.842	0.01 (Small)	----
MP	0.12 ± 0.03	0.11 ± 0.02	-5.76%	0.1 ± 0.01	-5.34%	1.087	0.349	0.062 (Medium)	----
Dec_max_	0.23 ± 0.08	0.2 ± 0.08	-12.55%	0.22 ± 0.09	11.39%	0.379	0.688	0.022 (Small)	----
Dec_total_	47.28 ± 2.93	47.54 ± 3.77	0.55%	46.16 ± 2.79	-2.88%	0.626	0.541	0.037 (Small)	----
Acc_max_	0.36 ± 0.15	0.32 ± 0.13	-10.57%	0.35 ± 0.13	7.46%	0.232	0.794	0.014 (Small)	----
Acc_total_	58.36 ± 4.79	58.52 ± 5.06	0.28%	57.02 ± 3.87	-2.56%	0.382	0.686	0.023 (Small)	----

TD: total distance, walking (from 0 to 7.2 km/h), jogging (from 7.2 to 14.4 km/h), running (from 14.4 to 20 km/h), HSR: high speed running (> 20 km/h), MP: metabolic power (W/kg), Dec_max_: maximum deceleration (Dec < -3 m/s^2^), Dec_total_: total deceleration distance, Acc_max_: maximum acceleration (Acc > 3 m/s^2^), Acc_total_: total acceleration distance, SD: standart deviation

**Fig. 1:**
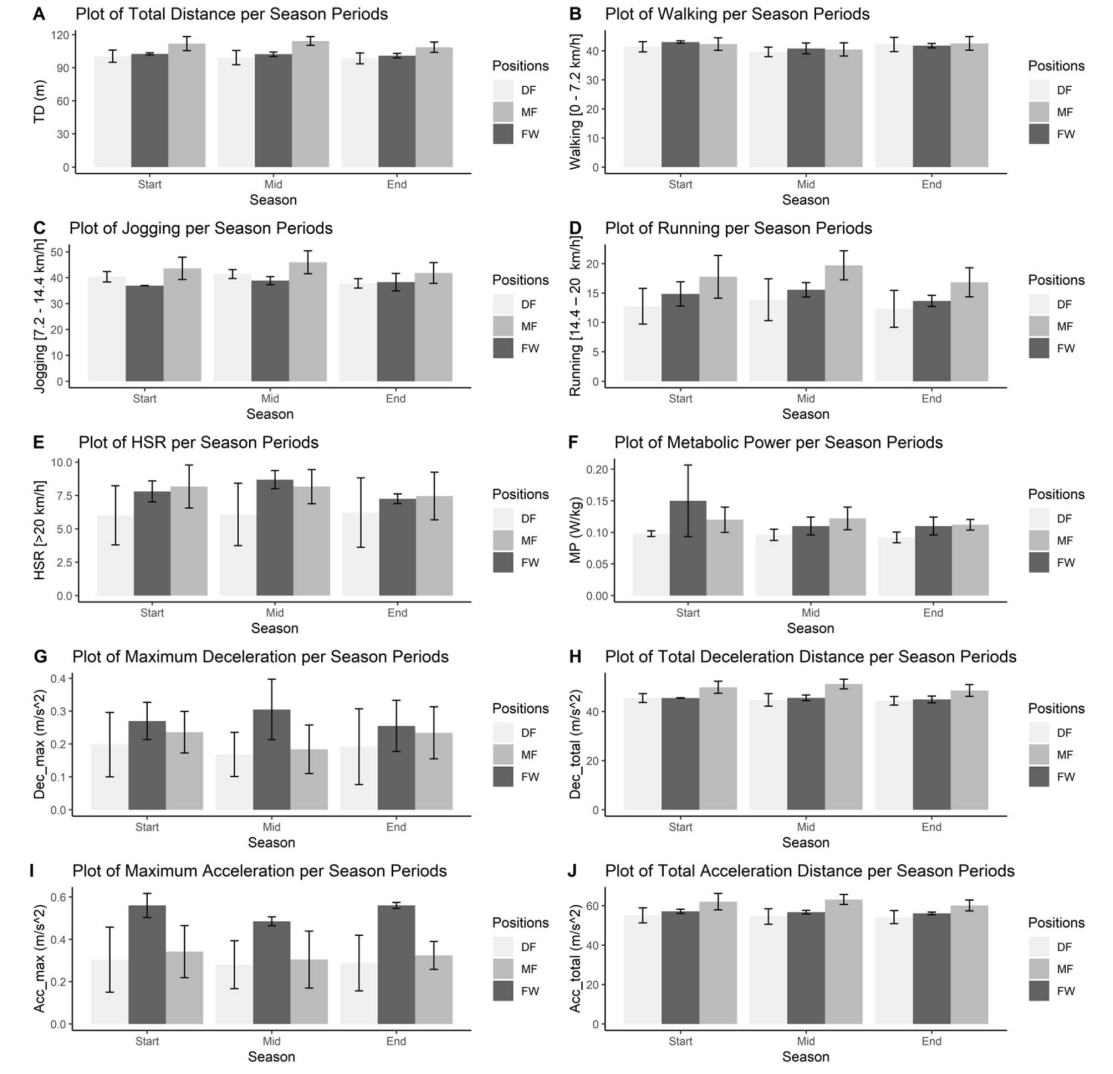
Match activities according to playing positions and season periods

**Fig. 2:**
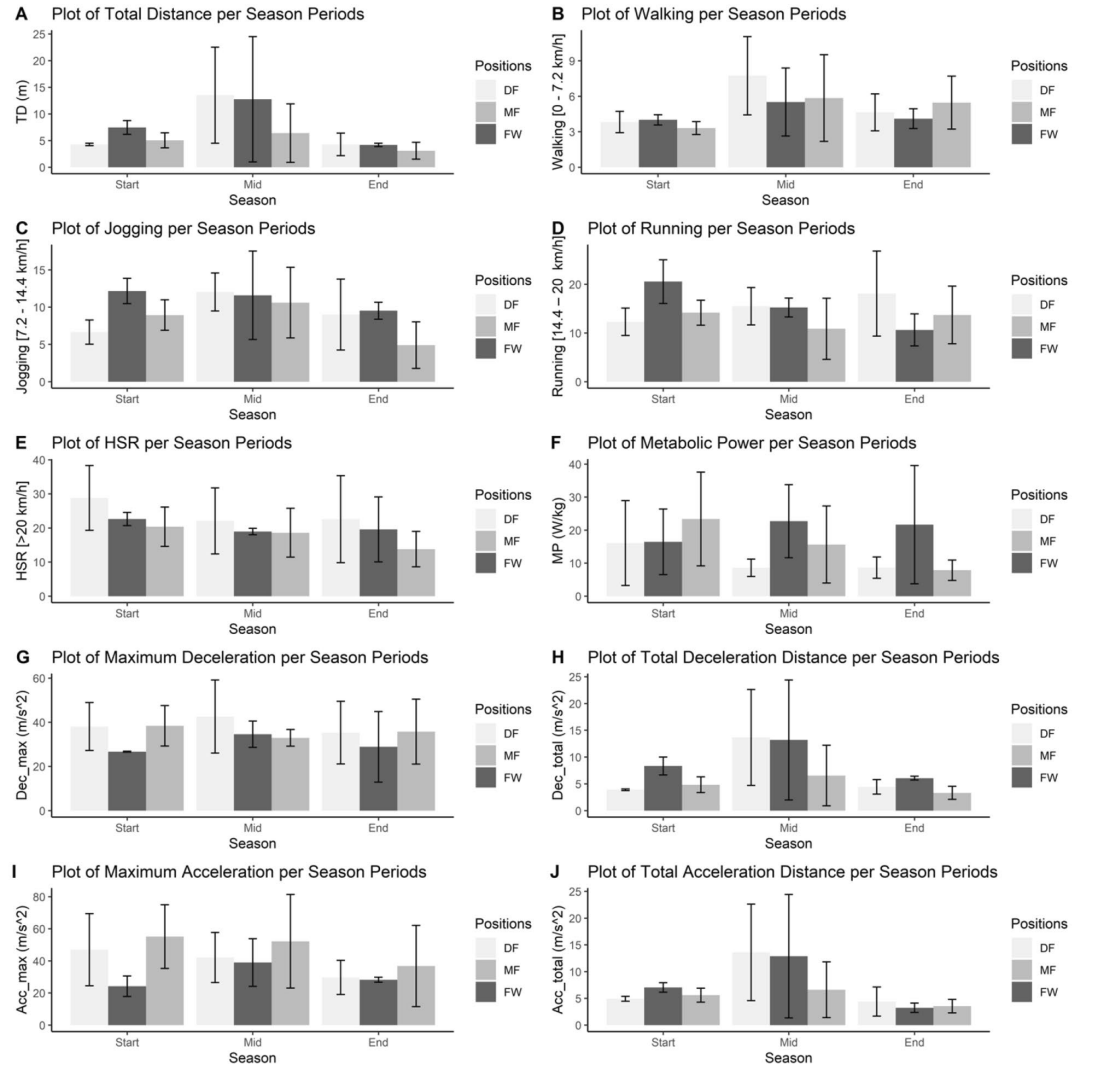
CV of match activities according to playing positions and season periods

The coefficient of variation (CV) for each performance metric is calculated by dividing the standard deviation of the parameter by its mean for each player to investigate the effect of the match-to-match variability. Mixed ANOVA analysis followed by Bonferroni post hoc test whenever applicable is conducted for the CV values of each parameter (Table 2). Bar plots are used to visualize the results (Figs. 1 and 2). P values less than 0.05 are considered significant. All the statistical analysis is conducted in the R programming language.

**Table 2:** Mean, Std. Deviations and Mixed ANOVA results for match activities according to playing positions and season periods

Variables (per min)	Season	DF	MF	FW	According to the Positions				According to the Season Periods			
					F	p	Effect Size	Source of difference	F	p	Effect Size	Source of difference
TD (m)	Start	100.55 ± 5.59	111.91 ± 6.37	102.65 ± 0.94	5.38	0.029	0.545 (Large)	DF < MF	1.03	0.399	0.025 (Small)	-
	Mid	99.28 ± 6.42	114.33 ± 3.96	102.31 ± 2.04	11.60	0.003	0.721 (Large)	DF < MF, FW < MF	7.80	0.013	0.204 (Large)	End < Mid
	End	98.57 ± 4.98	108.67 ± 4.69	101.01 ± 2.03	6.28	0.020	0.583 (Large)	DF < MF	0.50	0.666	0.245 (Large)	-
Walking [0–7.2 km/h]	Start	41.42 ± 1.78	42.36 ± 2.16	43.05 ± 0.40	0.63	0.553	0.123 (Medium)	-	17.80	0.001	0.266 (Large)	Mid < Start, Mid < End
	Mid	39.66 ± 1.63	40.49 ± 2.25	40.83 ± 1.85	0.35	0.712	0.073 (Medium)	-	9.98	0.007	0.176 (Large)	Mid < Start, Mid < End
	End	42.2 ± 2.43	42.56 ± 2.34	41.79 ± 0.75	0.09	0.916	0.019 (Small)	-	1.45	0.408	0.543 (Large)	-
Jogging [7.2–14.4 km/h]	Start	40.34 ± 2.03	43.6 ± 4.31	36.91 ± 0.05	4.31	0.049	0.432 (Large)	FW < MF	10.30	0.006	0.45 (Large)	End < Mid
	Mid	41.43 ± 1.72	45.95 ± 4.44	38.86 ± 1.56	4.35	0.048	0.492 (Large)	FW < MF	11.50	0.004	0.165 (Large)	End < Mid
	End	37.82 ± 1.83	41.81 ± 4.03	38.29 ± 3.39	2.19	0.168	0.327 (Large)	-	0.31	0.762	0.224 (Large)	-
Running [14.4–20 km/h]	Start	12.75 ± 3.02	17.76 ± 3.65	14.85 ± 2.05	3.02	0.099	0.401 (Large)	-	9.04	0.009	0.049 (Small)	End < Mid
	Mid	13.87 ± 3.56	19.7 ± 2.47	15.55 ± 1.23	5.14	0.320	0.533 (Large)	-	9.73	0.007	0.175 (Large)	End < Mid
	End	12.31 ± 3.14	16.82 ± 2.47	13.66 ± 0.96	3.64	0.070	0.447 (Large)	-	5.65	0.150	0.356 (Large)	-
HSR [> 20 km/h]	Start	6.01 ± 2.22	8.17 ± 1.61	7.8 ± 0.78	1.83	0.215	0.289 (Large)	-	0.56	0.591	0.002 (Nonsig.)	-
	Mid	6.07 ± 2.34	8.16 ± 1.28	8.69 ± 0.68	2.34	0.152	0.342 (Large)	-	1.32	0.319	0.053 (Small)	-
	End	6.22 ± 2.61	7.46 ± 1.79	7.26 ± 0.36	0.47	0.641	0.094 (Medium)	-	21.50	0.044	0.635 (Large)	End < Mid
MP (W/kg)	Start	0.10 ± 0.01	0.12 ± 0.02	0.15 ± 0.06	5.70	0.047	0.451 (Large)	DF < FW	1.56	0.269	0.121 (Medium)	-
	Mid	0.10 ± 0.01	0.12 ± 0.02	0.11 ± 0.01	4.23	0.051	0.484 (Large)	-	0.89	0.446	0.081 (Medium)	-
	End	0.09 ± 0.01	0.11 ± 0.01	0.11 ± 0.01	6.55	0.018	0.593 (Large)	DF < MF, DF < FW	1.78	0.360	0.372 (Large)	-
Dec_max_ (m/s2)	Start	0.20 ± 0.10	0.24 ± 0.06	0.27 ± 0.06	0.65	0.546	0.126 (Medium)	-	1.00	0.410	0.023 (Small)	-
	Mid	0.17 ± 0.07	0.18 ± 0.08	0.31 ± 0.10	2.65	0.125	0.370 (Large)	-	2.06	0.190	0.121 (Medium)	-
	End	0.19 ± 0.11	0.23 ± 0.08	0.26 ± 0.08	0.39	0.686	0.080 (Medium)	-	4.16	0.194	0.13 (Medium)	-
Dec_total_ (m/s2)	Start	45.43 ± 1.81	49.85 ± 2.49	45.47 ± 0.11	6.72	0.016	0.599 (Large)	DF < MF, FW < MF	1.15	0.365	0.058 (Small)	-
	Mid	44.71 ± 2.56	51.15 ± 1.94	45.55 ± 1.14	11.90	0.003	0.726 (Large)	DF < MF, FW < MF	9.84	0.007	0.216 (Large)	End < Mid
	End	44.32 ± 1.75	48.52 ± 2.40	44.88 ± 1.37	5.83	0.024	0.564 (Large)	DF < MF	0.59	0.629	0.143 (Large)	-
Acc_max_ (m/s2)	Start	0.30 ± 0.15	0.34 ± 0.12	0.56 ± 0.06	4.77	0.011	0.381 (Large)	DF < FW	0.21	0.816	0.007 (Nonsig.)	-
	Mid	0.28 ± 0.11	0.31 ± 0.14	0.49 ± 0.02	2.30	0.156	0.338 (Large)	-	0.27	0.772	0.024 (Small)	-
	End	0.29 ± 0.13	0.33 ± 0.06	0.56 ± 0.01	5.74	0.025	0.561 (Large)	DF < FW, MF < FW	3.00	0.250	0.661 (Large)	-
Acc_total_ (m/s2)	Start	55.11 ± 3.82	62.07 ± 4.16	57.18 ± 1.04	4.34	0.480	0.491 (Large)	-	0.82	0.475	0.012 (Small)	-
	Mid	54.57 ± 3.93	63.17 ± 2.55	56.77 ± 0.90	9.78	0.006	0.685 (Large)	DF < MF, FW < MF	6.13	0.024	0.157 (Large)	End < Mid
	End	54.25 ± 3.33	60.15 ± 2.78	56.14 ± 0.66	5.28	0.030	0.540 (Large)	DF < MF	0.48	0.674	0.317 (Large)	-

TD: total distance, HSR: high speed running, MP: metabolic power, Decmax: maximum deceleration, Dectotal: total deceleration distance, Accmax: maximum acceleration, Acctotal: total acceleration distance, DF: defenders, MF: midfielders, FW: forwards

## Results

One-Way ANOVA results for match performance metrics for different cycles is given in Table 3. The source of difference column denotes the Bonferroni test results when a significant difference is detected with one-way ANOVA. Effect sizes are also reported in Table 3.

Considering all measures and the overall participants, almost no significant differences in different period of the seasons were found apart from walking per minute. In this measure, middle of the season has significantly lower averages compared to start of the season [p = 0.021, %95 CI = (0.029; 3.79)] and end of the season [p = 0.011, %95 CI = (-3.99; -0.175)]

**Table 3:** Mean, Std. Deviations and Mixed ANOVA results for CV of match activities according to playing positions

Variables	Season	DF	MF	FW	According to the Positions				According to the Seasons			
					F	p	Effect Size	Source of difference	F	p	Effect Size	Source of difference
TD (m)	Start	4.28 ± 0.23	5.05 ± 1.4	7.47 ± 1.29	6.77	0.016	0.601 (Large)	DF < FW, MF < FW	4.84	0.042	0.453 (Large)	Start < Mid, End < Mid
	Mid	13.52 ± 9.02	6.39 ± 5.5	12.75 ± 11.75	1.08	0.380	0.193 (Large)	-	1.08	0.385	0.165 (Large)	-
	End	4.29 ± 2.11	3.10 ± 1.58	4.19 ± 0.31	0.63	0.553	0.123 (Medium)	-	0.93	0.519	0.348 (Large)	-
Walking [0–7.2 km/h]	Start	3.82 ± 0.90	3.31 ± 0.55	4.01 ± 0.44	0.95	0.423	0.174 (Large)	-	5.62	0.030	0.429 (Large)	Start < Mid, End < Mid
	Mid	7.74 ± 3.31	5.86 ± 3.67	5.52 ± 2.88	0.50	0.625	0.099 (Medium)	-	1.40	0.302	0.199 (Large)	-
	End	4.64 ± 1.56	5.46 ± 2.24	4.11 ± 0.83	0.46	0.643	0.093 (Medium)	-	0.51	0.664	0.236 (Large)	-
Jogging [7.2–14.4 km/h]	Start	6.65 ± 1.62	8.93 ± 2.04	12.16 ± 1.7	6.72	0.016	0.599 (Large)	DF < FW	4.53	0.048	0.366 (Large)	Start < Mid
	Mid	12.03 ± 2.54	10.61 ± 4.73	11.59 ± 5.93	0.16	0.857	0.034 (Small)		2.90	0.113	0.372 (Large)	-
	End	9.00 ± 4.75	4.90 ± 3.12	9.52 ± 1.14	1.83	0.216	0.289 (Large)		0.56	0.639	0.164 (Large)	-
Running [14.4–20 km/h]	Start	12.30 ± 2.82	14.17 ± 2.57	20.57 ± 4.48	5.64	0.026	0.556 (Large)	DF < FW, MF < FW	1.73	0.237	0.175 (Large)	-
	Mid	15.50 ± 3.85	10.87 ± 6.28	15.24 ± 1.94	1.24	0.335	0.216 (Large)		0.50	0.622	0.09 (Medium)	-
	End	18.09 ± 8.74	13.72 ± 5.91	10.64 ± 3.29	0.93	0.430	0.171 (Large)		3.14	0.242	0.74 (Large)	-
HSR [> 20 km/h]	Start	28.83 ± 9.50	20.38 ± 5.78	22.63 ± 1.94	1.67	0.242	0.271 (Large)	-	1.06	0.390	0.092 (Medium)	-
	Mid	22.09 ± 9.70	18.62 ± 7.16	18.95 ± 0.95	0.26	0.778	0.054 (Small)	-	1.29	0.328	0.205 (Large)	-
	End	22.61 ± 12.78	13.81 ± 5.20	19.58 ± 9.55	1.04	0.393	0.187 (Large)	-	0.35	0.741	0.139 (Large)	-
MP (W/kg)	Start	16.10 ± 12.86	23.39 ± 14.21	16.48 ± 9.93	0.43	0.062	0.088 (Medium)	-	1.80	0.226	0.203 (Large)	-
	Mid	8.60 ± 2.63	15.65 ± 11.68	22.72 ± 11.08	2.01	0.189	0.309 (Large)	-	2.71	0.127	0.302 (Large)	-
	End	8.66 ± 3.23	7.85 ± 3.07	21.67 ± 17.9	3.79	0.040	0.430 (Large)	DF < FW, MF < FW	0.11	0.904	0.076 (Medium)	-
Dec_max_ (m/s2)	Start	38.08 ± 10.86	38.41 ± 9.14	26.71 ± 0.19	1.24	0.335	0.216 (Large)	-	0.67	0.540	0.054 (Small)	-
	Mid	42.60 ± 16.54	32.95 ± 3.77	34.60 ± 5.95	0.95	0.424	0.174 (Large)	-	0.28	0.765	0.056 (Small)	-
	End	35.32 ± 14.20	35.79 ± 14.69	28.88 ± 16	0.18	0.842	0.037 (Small)	-	0.26	0.796	0.186 (Large)	-
Dec_total_ (m/s2)	Start	3.90 ± 0.15	4.83 ± 1.48	8.34 ± 1.68	11.00	0.004	0.71 (Large)	DF < FW, MF < FW	5.35	0.079	0.478 (Large)	-
	Mid	13.67 ± 8.96	6.55 ± 5.65	13.19 ± 11.2	1.12	0.369	0.199 (Large)	-	0.97	0.384	0.155 (Large)	-
	End	4.43 ± 1.36	3.32 ± 1.21	6.06 ± 0.36	4.74	0.046	0.454 (Large)	MF < FW	0.76	0.568	0.293 (Large)	-
Acc_max_ (m/s2)	Start	46.99 ± 22.47	55.14 ± 19.84	24.20 ± 6.39	1.70	0.237	0.274 (Large)	-	2.84	0.117	0.188 (Large)	-
	Mid	42.13 ± 15.58	52.23 ± 29.14	38.98 ± 14.87	0.36	0.707	0.074 (Medium)	-	3.52	0.130	0.114 (Medium)	-
	End	29.69 ± 10.66	36.8 ± 25.29	28.27 ± 1.6	0.25	0.784	0.053 (Small)	-	1.01	0.498	0.468 (Large)	-
Acc_total_ (m/s2)	Start	4.91 ± 0.46	5.60 ± 1.31	7.04 ± 0.89	5.43	0.048	0.433 (Large)	DF < FW	4.35	0.043	0.429 (Large)	End < Mid, Start < Mid
	Mid	13.60 ± 9.01	6.61 ± 5.22	12.88 ± 11.54	1.07	0.383	0.192 (Large)		1.07	0.387	0.166 (Large)	-
	End	4.39 ± 2.72	3.55 ± 1.26	3.24 ± 0.87	0.33	0.730	0.067 (Medium)		1.24	0.446	0.411 (Large)	-

TD: total distance, HSR: high speed running, MP: metabolic power, Decmax: maximum deceleration, Dectotal: total deceleration distance, Accmax: maximum acceleration, Acctotal: total acceleration distance, DF: defenders, MF: midfielders, FW: forwards

Mixed ANOVA test results for match performance metrics for different cycles of the season among each position are given in Table 1. The source of difference column denotes the Bonferroni test results when a significant difference is detected with mixed ANOVA. Table 3 also contains effect sizes.

TD: total distance, HSR: high speed running, MP: metabolic power, Dec_max_: maximum deceleration, Dec_total_: total deceleration distance, Acc_max_: maximum acceleration, Acc_total_: total acceleration distance, DF: defenders, MF: midfielders, FW: forwards.

TD: total distance, HSR: high speed running, MP: metabolic power, Dec_max_: maximum deceleration, Dec_total_: total deceleration distance, Acc_max_: maximum acceleration, Acc_total_: total acceleration distance, DF: defenders, MF: midfielders, FW: forwards.

At the start of the season, defenders have significantly lower total distance values compared to the midfielders [p = 0.011; d = -1.90 – Large, %Ch = -10.16%, 95% CI = (1.38; 21.4)]. At the middle of the season midfielders have higher total distance values on average compared to both defenders [p = 0.001; d = -2.82 – Large, %Ch = 15.16%, 95% CI = (6.09; 24.0)]. and forwards [p = 0.02; d = -3.81 – Large, %Ch = 11.73%, 95% CI = (0.156; 23.9)]. For the end of the season defenders have significantly lower total distance compared to the midfielders [p = 0.007; d = -2.09 – Large, %Ch = -9.31%, 95% CI = (1.97; 18.2)]. Walking per minute, running per minute and high-speed running per minute don’t differentiate among positions at any stage of the season. Midfielders have higher jogging per minute than forwards just in the start [p = 0.033; d = -2.19 - Large, %Ch = 18.13%, 95% CI = (0.15; 14.1)] and middle of the season [p = 0.027; d = -2.13 - Large, %Ch = -15.43%, 95% CI = (0.24; 14.6)]. In terms of metabolic power, all three positions have similar averages at the middle season, but defenders have lower averages than forwards both in the start [p = 0.026; d = -1.30 - Large, %Ch = -34.67%, 95% CI = (0.05; 0.106)] and end of the season [p = 0.043; d = -1.55 - Large, %Ch = -16.37%, 95% CI = (0.01; 0.0395)]. No differences are detected between the positions for maximum deceleration in any phase during the season. But for the total deceleration, midfielders have higher averages compared to the defenders in start of the season [p = 0.007; d = -2.03 - Large, %Ch = 9.71%, 95% CI = (0.792; 8.03)], middle of the season [p = 0.001; d = -2.83 - Large, %Ch = 14.41%, 95% CI = (2.59; 10.3)] and end of the season [p = 0.009; d = -2.00 - Large, %Ch = -9.49%, 95% CI = (0.615; 7.80)] whereas midfielders also have higher averages compared to forwards at the start of the season [p = 0.003; d = -2.48 - Large, %Ch = 9.60%, 95% CI = (0.421; 9.16)] and at the middle of the season [p = 0.013; d = -3.52 - Large, %Ch = 12.31%, 95% CI = (0.519; 10.7)]. For the maximum acceleration no significant differences were found among positions at the middle of the season. Still, defenders have lower averages than forwards at the start of the season [p = 0.046; d = -2.21- Large, %Ch = -45.71%, 95% CI = (0.0535; 0.566)] while midfielders only have lower values than forwards at the end of the season [p = 0.018; d = 4.96 - Large, %Ch = -42.14%, 95% CI = (-0.465; -0.007)]. For total acceleration, midfielders have higher averages compared to both defenders [p = 0.001; d = -2.60 - Large, %Ch = 15.78%, 95% CI = (3.07; 14.2)] and forwards [p = 0.0374; d = -3.35 - Large, %Ch = 11.28%, 95% CI = (1.23; 9.54)] at the middle of the season and midfielders have higher averages compared to the defenders at the end of the season [p = 0.011; d = -1.92 - Large, %Ch = 10.87%, 95% CI = (0.778; 11.0)].

Table 1 also gives the Mixed Anova and Bonferroni test results by focusing on the season cycle. For total distance per minute, defenders and forwards don’t show any significant differences in different processes whereas midfielders have a higher total distance per minute at the middle of the season compared to the end of the season [p = 0.049; d = 0.454- Small, %Ch = -4.94%, 95% CI = (2.97; 14.3)]. For walking per minute, while forwards don’t show any difference among cycles, both defenders and midfielders have lower walking per minute averages at the middle process compared to both the start [DF: p = 0.015; d = -1.03 - Large, %Ch = -4.25%, 95% CI = (1.58; 0.10); MF: p = 0.02; d = -0.846 - Large, %Ch = -4.40%, 95% CI = (1.93; 5.66)) and at the end of the season (DF: p = 0.032; d = 1.23- Large, %Ch = -6.04%, 95% CI = (-5.86; -1.72); MF: p = 0.04; d = 0.902 - Large, %Ch = -4.86%, 95% CI = (-5.89; -0.79)]. Forwards jogging per minute and running per minute performances don’t differentiate in different cycles of the season whereas defenders and midfielders have lower averages at the end of the season compared to the middle of the season for both jogging per minute [DF: p = 0.025; d = -2.02 - Large, %Ch = -8.70%, 95% CI = (0.455; 6.75); MF: p = 0.04; d = -0.98 - Large, %Ch = -9.01%, 95% CI = (3.06; 11.3)) and running per minute (DF: p = 0.043; d = -0.466 - Small, %Ch = -11.27%, 95% CI = (3.92; 7.05); MF: p = 0.019; d = -1.17 - Large, %Ch = -14.63%, 95% CI = (2.04; 7.80)]. For high-speed running, the only position that shows the difference is the forwards, in which the average at the middle of the season is higher than the end of the season [FW: p = 0.040; d = -2.64 - Large, %Ch = 19.77%, 95% CI = (1.22; 4.09)]. For performance metrics, metabolic power per min, maximum deceleration and maximum acceleration, no position showed significant differences among season periods. For deceleration total [MF: p = 0.049; d = -1.20 - Large, %Ch = 5.41%, 95% CI = (1.23; 6.49)] and acceleration total [MF: p = 0.048; d = -1.13 - Large, %Ch = 5.03%, 95% CI = (2.44; 8.50)], midfielders have significantly higher values at the middle of the season compared to end of the season. Results in Table 1 can be visually confirmed with the bar charts in Fig. 1.

The mixed ANOVA and Bonferroni test results and descriptive statistics for the coefficient of variation of the performance parameters per minute for each season period among positions are given in Table 2. Even after controlling match-to-match variability for individual players with the coefficient of variation, there are still differences among positions. For total distance per minute, both defenders [p = 0.005; d = -3.45 - Large, %Ch = -42.68%, 95% CI = (0.763; 5.61)] and midfielders [p = 0.021; d = 1.80 - Large, %Ch = -32.45%, 95% CI = (-4.85; -0.793)] have significantly lower averages compared to forwards just at the start of the season. No significant differences are detected at walking and high-speed running per minute. In jogging per minute, defenders have lower averages than forwards at the start of the season [p = 0.005; d = -3.32 - Large, %Ch = -45.35%, 95% CI = (1.24; 9.78)], and in running per minute, both defenders [p = 0.008; d = -2.21 - Large, %Ch = -40.17%, 95% CI = (1.37; 15.2)] and midfielders [p = 0.029; d = 1.75 - Large, %Ch = -31.11%, 95% CI = (-13.3; -0.493)] have lower averages than forwards at the start of the season.

For metabolic power per minute, both defenders [p = 0.044; d = -1.01 - Large, %Ch = -60.05%, 95% CI = (2.56; 28.6)] and midfielders [p = 0.035; d = -1.08 - Large, %Ch = -63.75%, 95% CI = (-29.4; -1.76)] have lower averages than forwards just for the end of the season. No significant differences are detected in any of the cycles for maximum deceleration and maximum acceleration. For total deceleration, forwards have significantly higher values compared to midfielders [p = 0.005; d = 2.22 - Large, %Ch = 72.57%, 95% CI = (-6.16; -0.85)] and defenders [p = 0.001; d = 3.73 - Large, %Ch = 113.61%, 95% CI = (1.78; 7.09)] at the start of the season whereas midfielders also have lower averages than forwards at the end of the season [p = 0.025; d = -3.08 - Large, %Ch = -45.29%, 95% CI = (-5.59; -0.10)]. For total acceleration, forwards have higher averages than defenders at the start of the season [p = 0.028; d = 3.00 - Large, %Ch = -30.19%, 95% CI = (0.143; 4.40)].

Table-3 gives the Mixed ANOVA results for the coefficient of variation values of the performance parameters by focusing on the season cycles for each position. After controlling match-to-match variability for individual players with the coefficient of variation, few differences are detected for the defenders. Defenders have higher averages at the middle of the season compared to the start of the season for the total distance per minute [p = 0.035; d = -1.45 - Large, %Ch = 215.69%, 95% CI = (-18.3; -0.211)], walking per minute [p = 0.028; d = -1.90 - Large, %Ch = 102.46%, 95% CI = (-7.59; -0.247)], and total acceleration [p = 0.020; d = -1.62 - Large, %Ch = 176.72%, 95% CI = (-17.9; -0.50)]. Defenders also have higher averages at the middle of the season compared to the end of the season for the total distance per minute [p = 0.015; d = 1.41 - Large, %Ch = 215.36%, 95% CI = (0.207; 18.3)], walking per minute [p = 0.023; d = -1.20 - Large, %Ch = 66.72%, 95% CI = (0.573; 6.77)], and total acceleration [p = 0.017; d = -1.38 - Large, %Ch = 209.78%, 95% CI = (0.025; 18.4)]. Also, for the defenders, in the jogging per minute, significantly higher averages are detected at the middle of the season compared to the start of the season [p = 0.043; d = 2.53 - Large, %Ch = 81.10%, 95% CI = (-10.9; -0.09)]. Results in Table 2 can be visually confirmed with the bar charts in Fig. 2.

## Discussion

As far as we know, this is the first study to compare the seasonal variations of matches through external load measures of a Turkish Super League team and to evaluate them according to different playing positions. In the literature, a recent study conducted on professional teams in Turkey Super League showed seasonal trends in technical variables. At the same time, there were no gradual changes in external load measures throughout the three seasons [14]. The present study revealed that midfielders fulfilled more external load demands throughout the season than other player positions. In addition, the external load measures of some playing positions showed changes according to different periods of a soccer season. It was seen that midfielders’ external load profile decreased at the end of the season compared to the middle of the season. Lastly, high variability was detected in some external measures according to different periods of the player and season.

The present study revealed that the total distance covered per minute by midfielders was significantly higher than other playing positions (forwards and defenders) during the match at all stages of the season. This result is in line with previous studies, which demonstrated that midfielders covered a greater total or relative distance (m/min) than defenders and forwards in professional soccer matches [6, 9], such as Australian Football League [17], English Premier League [18], and Spanish First Division [19]. The fact that midfielders covered more total distance during the match compared to other positions can be explained by their better-developed intermittent endurance capacity and higher VO_2max_ values in professional soccer [20]. Also, previous studies have reported conflicting results in comparing the external load across player positions throughout the season [6–8]. The divergent findings can be attributed to a variety of factors, such as the strategic preferences and game strategies employed by coaches, variations in competitiveness across different leagues, and the utilisation of diverse analytical approaches (including accumulated data, monotony, and/or strain indices) when interpreting the data [18, 21].

Moreover, the present study showed that midfielders had significantly higher total acceleration and deceleration distances than defenders in almost every season, and also attackers in some periods. Consistent with our results, it was noted by previous studies on elite soccer players that midfielders had a greater frequency of very high-intensity accelerations and decelerations higher than attackers [22] and defenders [2] due to the limited space available within central areas of the pitch, especially for central midfielders [23]. It has been suggested that the higher number of acceleration and deceleration events in midfielders was due to their specific role in the game relative to their playing style [19]. Regarding metabolic power, which is a kinematics-based approach and, is used to estimate the energy expenditure of acceleration and deceleration during interval running in team sports, including soccer [11, 17, 24], the present study revealed that attackers and midfielders presented significantly higher values than the defenders at the beginning and end of the season. This result was compatible with the survey carried out by Alcantarilla-Pedrosa [25], which remarked that metabolic power and total energy consumption of midfielders and forwards during match-play were higher than other positions, especially defenders in Australian football players. These results reflect the higher intensity activity volume of midfielders in-game than offensive players during the competitive season.

Considering the results of the studies and explanations mentioned above, in our research, midfielders displayed higher values in external load measures (total distance covered, metabolic power, acceleration and deceleration etc.) per minute compared to other playing positions, especially defenders during the competitive soccer season. This result may be explained by the physical/physiological demands and responsibilities of the position played by midfielders [6, 17]. According to this, midfielders are the players who stand out in the formation of offence in most tactical squads and have a high activity profile. At the same time, they v out repetitive backwards and forwards between the attack and eeeeeee. Also, the most crucial point that the coaches should pay attention to in the present study is that all external load measures were lower for defenders than for other playing positions during the season. This result may need to be more accurate in the training load for defenders [8]. Therefore, coaches should compensate for the external load gap between defenders and midfielders during training, and to increase the external intensity profile of the defenders.

Specifically, in our study, midfield players exhibited the highest external load profile per minute during the mid-season period compared to other playing positions. Consistent with our results, Ponce-Bordon et al. [26] discovered that teams exhibited a higher total distance covered and engaged in more sprints during the middle of the season. This aligns with the notion of teams adapting to the training load, and competition demands as the season advances from the early season to the mid-season phase compared to other periods of the season. The observed increase in physical performance indicators suggests that teams undergo adjustments and improvements in conditioning and tactical strategies over the season [27]. Furthermore, the current study demonstrated a significant decrease in the mentioned external load measures of midfield players towards the end of the season compared to the mid-season. Similarly, the recent survey conducted on consecutive matches (n = 1520) of four seasons in Spain’s La Liga, utilizing an optical tracking system, indicated that the total distance covered decreased from mid-season to the end regardless of the player’s position in the game.

Furthermore, the decline in external load towards the end of the season can be attributed to the higher workload experienced by midfielders at the beginning and middle stages of the season, altered training strategies or changes in tactical approaches. For instance, in our study, the noticeable increase in walking distance per minute observed among midfield players towards the end of the season could be attributed to accumulated fatigue. This finding suggests that midfielders may experience a higher level of physical fatigue as the season progresses, decreasing their overall external load. Studies in the literature report the accumulation of fatigue and a subsequent decrease in physical performance towards the end of the season without making a distinction based on player positions. For example, recent studies have stated that fatigue accumulated during the season and subsequent incomplete recovery processes might lead to a decline in physical performance towards the end of the season due to the difficulty of maintaining physical fitness levels [28, 29]. Regarding the playing positions, Nobari et al. [30] supported our interpretation about accumulated fatigue at the end of the season period for midfielders by showing that the most hooper index changes between mesocycles of a soccer season were more dramatically related to midfielders than to other positions. In addition, when compared to the mid-season period, muscle soreness and fatigue values ​​of midfielders at the end of the season period were found to be higher than other playing positions. The same study observed variations between playing positions in high-intensity parameters rather than low-intensity-related parameters at the end of the season period. It seems that the frequency of explosive eccentric movements, such as high-intensity acceleration and deceleration, performed during a match can result in decreased neuromuscular performance capacity and increased indicators of muscle damage at the end of the season, particularly in midfielders, which may trigger an increased risk of injury [2]. Based on the findings, coaches should implement specific training plans aimed at maintaining and enhancing the cardiovascular fitness and high-intensity profile of players, particularly midfielders, throughout all phases of the season, including the end of the season. These training plans should address the unique demands of midfielders and focus on strategies to manage and minimize fatigue accumulation. By providing targeted conditioning and workload management, coaches can optimize the performance and mitigate the potential decline in physical capacity for midfielders compared to players in other positions [9, 30].

Some decisive external load variables or playing positions over a season may differ from one match to the next. In our study, in general, high variability was found in external measures related to high-intensity attackers’ activities in matches at the beginning and end of the season. In addition, the highest variability in walking, jogging and total acceleration was seen in mid-season compared to other periods in defenders. In a study supporting our findings, Martin-Garcia et al. [31] pointed out that the external load in micro-cycles varied greatly depending on the tactical role of the players in the team. Besides this, positional variability in match activity profiles may be due to contextual factors frequently cited in recent studies. Accordingly, our study revealed that high variability activity patterns (seasonal fluctuations) observed at different season periods, especially in forwards and defenders, underline the need for a more personalized approach to monitoring external load for these playing positions [15].

The present study contains some limitations. Firstly, the specified external load variables determined by analyzing the matches played by only one team in the Turkish Super League during a season were obtained. Secondly, video tracking systems are the most widely used in the analysis of the physical demands of the players. However, unlike these systems, the development of GPS technology has recently gained ground. As GPS technology consists of more sensitive devices, it can provide more accurate and consistent results in controlling the load in training and matches, especially data on acceleration and deceleration. This should be taken into account in future studies [25]. Third, in our research, because of 250 individual match analysis, playing positions were evaluated in three groups (defender, midfielder and attacker). In future studies, the number of player positions can be increased (for example, a classification can be made as central or wide for both defenders and midfielders), as there are differences in external measures between players playing in the defensive or midfield area. However, it is also suggested using larger sample sizes. As explained above, considering that contextual factors, such as match status, match location (i.e. local vs. visitor), outcome (i.e. win vs. lose), and the quality or level of the opponent [9] is effective on external load measures. Still, they were not evaluated in this study which may be considered the last limitation. Lastly, the study’s limitations are that there is an unequal number of matches between observed periods. Also, the observed season was during the COVID pandemic, and this could impact the results significantly. However, since the team in the study is a high-level professional team, the results are in parallel with the studies in the literature, as the athletes continue their training during the Covid-19 period. To generalize the results, it is recommended to increase studies with similar designs and larger sample groups (more teams) in the same league or in different leagues, considering the gender and age factor.

These findings have practical implications for coaches, sports scientists, and performance analysts in soccer. Firstly, recognizing the lower external load demands on defenders provides insights into their specific role within the team. Coaches can adjust training programs to focus on skills and tactics that are crucial for defenders, such as positioning, tackling, and anticipation, rather than emphasizing high-intensity physical conditioning. Moreover, understanding the higher activity profiles of midfielders at the beginning and middle of the season can inform training strategies. Coaches can design training sessions that replicate the demands experienced by midfielders during matches, ensuring that they are adequately prepared for the physical challenges they encounter. However, it is important to consider the decreasing activity profiles of midfielders towards the end of the season. Coaches can modify training loads and implement recovery strategies during this period to manage fatigue and optimize player performance. Additionally, the findings highlight the importance of periodization in training plans. By considering the observed changes in activity profiles across the season, coaches can structure training phases to align with the specific needs of each player position. This approach allows for tailored training and workload management, aiming to optimize player performance and minimize the risk of injuries.

The clinical significance of these findings has important implications for clinicians, players, and researchers involved in soccer or sports medicine. For clinicians, understanding the external load demands placed on different player positions provides valuable information for injury prevention and player management. By recognizing the variations in physical activity between positions, clinicians can tailor their rehabilitation programs, training regimens, and injury prevention strategies accordingly. For example, since midfielders are engaged in more intense physical activities, they may be at a higher risk of certain injuries or fatigue-related conditions. Clinicians can use this knowledge to develop targeted prevention strategies and specific training interventions to mitigate these risks and optimize player performance.

## Conclusions

The present study yielded important findings regarding the external load demands placed on different player positions in soccer. Specifically, the research revealed that defenders consistently exhibited the lowest values in all external load measures during matches, indicating relatively lower physical activity compared to players in other positions. In contrast, midfielders displayed a higher activity profile at the beginning and middle of the season in comparison to other positions. This implies that midfielders are involved in more intense physical activities, such as covering large distances, engaging in frequent high intensity activities, and participating in offensive and defensive transitions. However, an interesting observation was made regarding the activity profiles of midfielders towards the end of the season. It was found that there were decreases in these profiles, indicating a reduced external load experienced by midfielders in the latter stages of the season. The observed decrease in the external load profile of midfielders towards the end of the season emphasizes the need for tailored training programs and workload management strategies. By considering these findings, monitoring external load measures, such as m/min, throughout the season is essential for evaluating the effectiveness of training programs and ensuring that players are adequately prepared for the physical demands of their positions. By regularly tracking and analyzing these metrics, coaches and sports scientists can gain valuable insights into players’ workload, fatigue levels, and readiness for matches. This information can guide adjustments in training plans, allowing for individualized load management, and reducing the risk of overtraining and injuries.

## Data Availability

The data presented in this study are available on website: https://osf.io/h96xy/ with Identifier: DOI 10.17605/OSF.IO/H96XY.
